# Suggestion without Sleep

**Published:** 1907-08-10

**Authors:** Edwin Ash

**Affiliations:** (Clinical Assistant, Great Northern Hospital, London). London, W.


### SUGGESTION WITHOUT SLEEP.

Sir,—Referring to Dr. Bryan's letter criticising my article on "Treatment by Suggestion without Sleep" (published in The Hospital of July 6), I should be greatly obliged if you would allow me space to make a few remarks anent this important subject. Having made many hundred experiments to investigate the processes we know as "Hypnotism" and "Suggestion," I have come to the conclusion that in hypnosis we have a mental state differing more essentially from the normal sleep state, and still more essentially from the normal waking state, than is generally allowed by modern writers on hypnotism. Moreover, I am forced to conclude from innumerable observations that the vast majority of experiments quoted and recorded as hypnotic experiments have really been experiments with simple suggestion. And I am ready to confess that I myself was for some time under a misapprehension similar to that which makes so many people confuse hypnotic experiments with experiments in simple suggestion. It is because the induction of hypnosis by suggestion is the method usually employed by those who practise therapeutic hypnotism at the present day that so many mistakes have been made in this connection. Consequently it is not yet generally understood that true hypnotism means somnambulism, or the dreamy state that precedes it. Now if Dr. Bryan will hypnotise a few willing people and carefully interrogate them at all stages of his experiments he will find that in all cases where actual hypnotic control has been obtained there is a sensation of temporary loss of power, and in the deeper stages of confused or lost consciousness to actual surroundings. Yet it is quite possible, and with many practitioners usual, to make use of suggestion in relieving symptoms or correcting morbid ideas without anything of this kind being experienced by the patients. Therefore I argue that unless one endeavours to obtain hypnotic control when giving suggestions, no hypnosis in the real sense of the word will be produced. I will go further and say that it is possible to induce a true natural sleep by suggestion, which is not a hypnotic sleep, firstly, because in it none of the characteristics of hypnosis will be present, and, secondly, because in such a sleep suggestions made by the operator have no effect.

What the exact difference between the two processes is I do not know, but I do know that the experience of what seems to be loss of contact with the mind of one's patient is not uncommon in using these methods. These patients are not hypnotised; they do not become somnambulic; neither are they in that indefinite comatose state which sometimes results from careless use of hypnotism, the state known as hypnotic "lethargy." And so it seems right to me that a definite distinction should be made between simple suggestion and hypnotism, especially as we know that hypnosis can be obtained by many methods other than suggestion. If Dr. Bryan will hypnotise a few people by Braid's original method, which did not admit the principle of suggestion, he will find that suggestions made to such subjects whilst in hypnosis have nothing like the effect that they would have had the subjects been hypnotised by suggestion. It is becoming more evident to me every day that it is simple direct suggestion, backed up by that personal factor—(acting as indirect suggestion) which has been so ably demonstrated by a well-known physician whose name I have not obtained permission to use—that constitutes one of the most important adjuncts we have to routine therapeutic measures. The fact that hypnosis can so often be produced by suggestion, and is then represented by a condition of greatly enhanced suggestibility, has led many enthusiasts into thinking that hypnotism is the basis of successful suggestive therapeutics. This matter is too far-reaching to be adequately discussed in a short letter, but I am glad to have this opportunity of stating my convictions with regard to it. Hypnotism has its uses in certain cases, but must be closely watched; suggestion as a powerful adjunct to treatment by other methods is ready to the hand of every practitioner who is wise enough to make himself conversant with its principles. And as there is a mental factor in every case of disease, so it is those practitioners that make use of suggestion in their work (often unknowingly) who get the best results and earn the deepest thanks of their patients.

I am, faithfully yours,

Edwin Ash,

(Clinical Assistant, Great Northern Hospital, London).

London, W., August 6.

